# Anatomy of the stemmata in the *Photuris* firefly larva

**DOI:** 10.1007/s00359-018-01312-2

**Published:** 2019-01-16

**Authors:** Frederick Murphy, Andrew Moiseff

**Affiliations:** Department of Physiology and Neurobiology, University of Connecticut, 75 N. Eagleville Road, Unit 3156, Storrs, CT 06269 USA

**Keywords:** *Photuris*, Firefly larva, Fusion stemmata, Holometabolous, Rhabdomeres

## Abstract

Fireflies (Coleoptera: Lampyridae) have distinct visual systems at different stages of development. Larvae have stemmata and adults have compound eyes. Adults use compound eyes to mediate photic communication during courtship. Larvae do not manifest this behavior, yet they are bioluminescent. We investigated the structure of stemmata in *Photuris* firefly larvae to identify anatomical substrates (i.e., rhabdomeres) conferring visual function. Stemmata were located bilaterally on the antero-lateral surfaces of the head. Beneath the ~ 130 µm diameter lens, we identified a pigmented eye-cup. At its widest point, the eye-cup was ~ 150 µm in diameter. The optic nerve exited the eye-cup opposite the lens. Two distinct regions, asymmetric in size and devoid of pigmentation, were characterized in stemmata cross-sections. We refer to these regions as *lobes*. Each lobe contained a rhabdom of a radial network of rhabdomeres. Pairs of rhabdomeres formed interdigitating microvilli contributed from neighboring photoreceptor cell bodies. The optic nerve contained 88 axons separable into two populations based on size. The number of axons in the optic nerve together with distinct rhabdoms suggests these structures were formed from ‘fusion stemmata.’ This structural specialization provides an anatomical substrate for future studies of visually mediated behaviors in *Photuris* larvae.

**Electronic supplementary material:**

The online version of this article (10.1007/s00359-018-01312-2) contains supplementary material, which is available to authorized users.

## Introduction

Vision, in holometabolous insects, can be mediated by fundamentally different visual organs—stemmata or compound eyes. Compound eyes and their underlying optic ganglia facilitate highly specialized and diverse behaviors (Briscoe and Chittka 2001; Somanathan et al. 2008; Katsov and Clandinin 2008; Farnier et al. 2015). The variation of visually mediated behaviors across adult insects is accomplished via a shared and conserved cellular paradigm (Nilsson 1989). The anatomical elements contained in compound eye ommatidia are conserved among the majority of insects: two primary pigment cells, four cone cells and eight photoreceptors (Melzer et al. 1997; Paulus 2000; Harzsch et al. 2005; Nilsson and Kelber 2007). Despite such cellular similarity, different optical strategies have evolved, often independently, to meet behaviorally specific needs influenced by an animal’s environmental niche, e.g., navigation, recognition of conspecifics, predator avoidance or prey detection [for a review see (Land 1997)]. The ubiquitous yet diverse compound eyes have made these visual systems appealing targets for studying the relationship of organ structure to function. However, compound eyes are not unique to all aspects of an insect’s life cycle. In holometabola (metamorphic insects) which comprise greater than 80% of known insect species (Kristensen 1999), compound eyes are predominantly restricted to the adult form. In pre-metamorphic holometabolous insect larvae, compound eyes are absent (except for some *Mecoptera* genera) (Melzer et al. 1994). In these larvae, vision is mediated by distinct eyes referred to as stemmata.

Stemmata are single-lensed eyes positioned bilaterally on the larval head. Morphological examination (Paulus 1986), later corroborated by molecular evidence (Liu and Friedrich 2004) revealed that stemmata evolved from compound eye ommatidia of hemimetabolous ancestors. Despite evolving from the highly conserved cellular plan of the compound eye, independent divergence has produced a variety of structural and functional specializations in stemmata [for a comprehensive review of stemmata see (Gilbert 1994)].

In beetles (Coleoptera), the order of holometabolous insects with the largest diversity of species, larvae have anywhere from zero to six stemmata on each side of their head, an arrangement that has been linked to specific functional advantages. This diversity of sensory specialization within the visual system facilitates the diverse behaviors seen amongst larvae. *Cicindela chinensis* (tiger beetle) have six stemmata on each side of the head, two of which are enlarged compared to the other four. Each stemma contains a single lens and is separated from neighboring stemmata. The two enlarged stemmata contribute a thick nerve bundle containing retinula cell axons and synapse in an underlying neuropil before terminating in the brain (Toh and Mizutani 1994). *Thermonectus marmoratus* (aquatic sunburst diving beetle) also have six stemmata, but located within the tubular shape of their dorsal stemmata are three separated retinas, an organization that is distinct from other known stemmata (Mandapaka et al. 2006). These different visual structures each facilitate specific, predatory visual behaviors. The tiger beetle neuropil contains motion-sensing neurons that mediate an ambushing predatory ‘jumping snap’ behavior as well as a ‘withdraw-escape’ response (Toh and Mizutani 1994), and the sunburst diving beetle eyes are optimized for predatory behaviors in aquatic environments (Buschbeck et al. 2007).

In contrast to multi-stemmata larval visual systems, fireflies, like all members of the *Elateroidea* superfamily, have a single stemma on each side of the head. Fireflies spend the majority of their lives in larval form, growing through multiple instars (McLean et al. 1972). *Photuris* fireflies, remain in larval form for 9–24 months, the latter requiring multiple overwintering periods (McLean et al. 1972). Firefly larvae, like adults, are bioluminescent, but unlike adults, they do not rely on their visual systems for the reception and processing of flash patterns (Carlson and Copeland 1985). In this study, we investigated the visual system of firefly larvae (*Photuris* genus).

The anatomy of firefly larval eyes and the behavioral significance of their visual system are unknown. We used various microscopy methods to investigate the structure of *Photuris* firefly larval stemmata (used interchangeably with ‘eye(s)’ in this report). Given beetle diversity (Zhang et al. 2018) and the breadth of anatomical solutions to mediate visual function found throughout Coleoptera, we propose that examining such specializations of the firefly larval eye will support our broader goal of elucidating ecologically relevant behaviors mediated by the firefly larval visual system.

## Materials and methods

### Firefly stocks

All firefly larvae (*Photuris* genus) were collected locally near Storrs, CT, September–November, 2016 and 2017. Larvae were kept in transparent containers with their natural soil and vegetation extracted from the site of collection and were maintained in the lab for up to 8 months. Containers were kept at room temperature until mid-November when they were transferred to a 4 °C environment to simulate overwintering and delay pre-mature pupation. The soil was kept moist and larvae were fed worms and slugs.

### Gross anatomy

Specimens were dissected in firefly Ringers (Carlson 1968). The cuticle of the head and pronotum were removed using the tip of a 25-gauge hypodermic needle as a microscalpel. This exposed the muscular tissue within the head. The lenses were removed from the remaining cuticle, and the entire head and thorax were subsequently fixed in 4% paraformaldehyde in 0.1M PBS at 4 °C, overnight. Images were acquired by an Olympus DP72 camera and Olympus CellSens imaging software. Images were processed, post-acquisition, using ImageJ 64. Figures were assembled using GIMP v2.10.

### Embedment for light and electron microscopy

Stemmata, the optic nerve and the brain were dissected from the larva in firefly Ringers. The optic nerve was transected and separated from the large nerve bundle that projected to the brain. The stemmata along with the optic nerve were removed from the surrounding cuticle (lenses were left intact for electron microscopy sections) and subsequently washed in 0.1M HEPES buffered solution. The tissue was fixed by immersion in a modified Karnovsky solution (2.5% glutaraldehyde, 2% paraformaldehyde, 0.1M HEPES buffer, pH 7.4) overnight at 4 °C. Tissue was subsequently washed three times (20 min each) in 0.1M HEPES pH 7.4 at room temperature and left overnight at 4 °C. Stemmata were post-fixed in a secondary solution (1% osmium tetroxide and 0.8% potassium ferricyanide in 0.1M HEPES buffer) for 1.5 h at 4 °C. Following three washes in 0.1M HEPES buffer, the tissue was dehydrated in a graded ethanol series (30–100%) and cleared in propylene oxide (2 times, 15 min each). Tissue was then infiltrated using an epoxy-based resin-embedding media (Eponate 812, DDSA, NMA and DMP-30 accelerator). The resin-embedded tissue was polymerized (60 °C oven) and prepared for sectioning.

### Osmium ethyl gallate-stained tissue

Stemmata to be stained with ethyl gallate were dissected in 0.1M PBS and fixed overnight at 4 °C (2.5% glutaraldehyde, 2% paraformaldehyde in 0.1M cacodylate buffer). Tissue was washed in 0.2M cacodylate buffer, pH 7.6, three times (10 min each) and post-fixed in 2% osmium tetroxide in 0.1M cacodylate buffer for 1.5 h. Tissue was washed in 0.2M cacodylate buffer (3 times, 15 min each) and left overnight in fresh 0.2M cacodylate buffer, 4 °C. The washed tissue was incubated in saturated ethyl gallate solution (Leise and Mulloney 1986) for 24 h. Following treatment with ethyl gallate, the tissue was washed two times in 10% acetone (10 min each) and embedded in an epoxy-based resin media as previously described.

### Light microscopy

Thin sections (2 µm) were cut using a glass knife in the longitudinal and transverse planes. Sections were subsequently incubated for ~ 20 s at 27 °C in azure II methylene blue stain for contrast. All images were acquired by an Olympus DP26 camera and Olympus CellSens imaging software. Brightness and contrast of displayed images were post-processed using ImageJ 64.

### Electron microscopy

Ultrathin sections (60–80 nm) of the stemmata (transverse and longitudinal plane) and the optic nerve (cross-section) were cut using a diamond knife. Longitudinal sections of the stemmata were collected on Formvar-coated slot grids or copper mesh grids. All cross-sections of the optic nerve were collected on copper mesh grids. After collection, tissue was heavy metal stained in 4% ethanolic uranyl acetate for 8 min followed by 2.5% modified Sato’s lead citrate (Sato 1968) for 3 min. Micrographs were obtained using a FEI Tecnai G2 Biotwin electron microscope operated at 80 kV. Digital images were acquired with an AMT XR40 4-Megapixel, side-mounted CCD camera. Micrographs were adjusted for brightness and contrast post-acquisition using Gimp v2.10.

### Confocal microscopy

Stemmata and the intact optic nerve were removed from the firefly larva in 1X PBS. Autofluorescent samples were immediately fixed in 4% formaldehyde at room temperature for 1 h and subsequently cover-slipped in VECTASHIELD mounting media (Vector Laboratories). Stemmata processed for retrograde labeling of the optic nerve had the cut end of the optic nerve incubated in the dark with 5 mg/ml solution of Dextran-Texas Red in 1X PBS (3000 MW, Invitrogen) for 1 h at room temperature. Samples were then transferred to 4 °C overnight. Following incubation, samples were fixed in 4% formaldehyde at room temperature for 1 h and cover-slipped in VECTASHIELD mounting media. All mounted samples were stored in the dark at 4 °C. Images were acquired using a Leica TCS SP8 confocal microscope.

### Statistical analysis

The area of the axons (Fig. 3a) was measured using FIJI (Schindelin et al. 2012). The distribution of area measurements of the optic nerve axons was tested for normality using the Anderson–Darling normality test. Decomposition of the distribution to detect the number of means within our sample data was performed using Mclust (Fraley and Raftery 2007). Means were detected and plotted with density curves using Mixtools (Benaglia et al. 2009).

## Results

### Firefly larva and stemmata

**Fig. 1:**
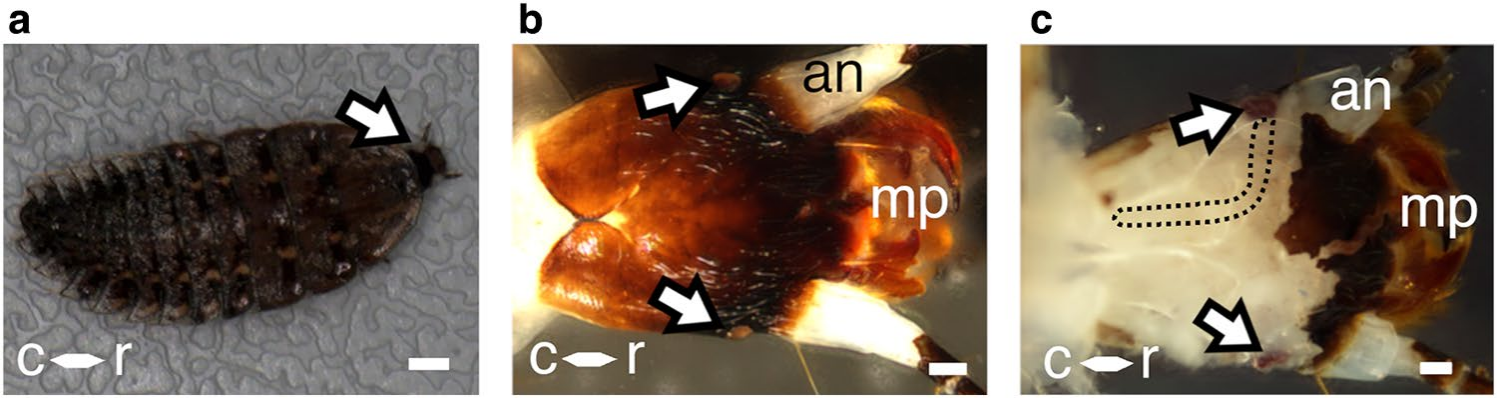
*Photuris* firefly larva. **a** Dorsal perspective. A retractable head extends (arrow) beneath the anterior cuticle segment, the pronotum. Scale = 1 mm. **b** Single-lensed bilateral stemmata (arrows). **c** Larval head with cuticle removed. Removal of the cuticle reveals the in situ position of each stemma (arrows). The optic nerve is not visible in ‘b,’ but its underlying orientation is outlined by the black dotted line. *mp* mouth parts, *an* antenna, *r* rostral, *c* caudal. Scales = 150 µm

In their larval form, *Photuris* fireflies measured ~ 1.5 cm from the tip of the anterior cuticular segment (known as the pronotum) to the tail (Fig. 1a). Rigid segments of cuticle tiled the dorsal surface of the animal’s body (Fig. 1a). A retractable head was located beneath the pronotum (black outlined arrow, Fig. 1a). The head contained mouth parts, antennae and the stemmata. The lens of each stemma was located posterior to the base of each antenna. On the exterior surface, the single lens of each stemma was ~ 130 µm in diameter (arrows, Fig. 1b). Removal of both the cuticle covering the head and each lens revealed the underlying stemmata (arrows, Fig. 1c). The stemmata, densely pigmented, contrasted against the pale surrounding tissue which primarily consisted of muscle fibers and trachea. Ventral to the exposed soft tissue, the stemma nerve exited the eye, opposite the lens, toward the mid line of the head capsule, curved and extended caudally (Fig. 1c). Although the optic nerve itself is not visible in Fig. 1c, the in situ orientation of the nerve is artificially indicated by the black dotted outline. The visual system and all connecting nerve fibers could be extracted from the surrounding tissue to reveal a single optic nerve fascicle that projected ipsilaterally from each eye for approximately 2.5 mm (Online Resource 1). The optic nerve converged into a single nerve bundle with two additional nerve tracts. This nerve bundle entered adjacent hemispheres of the brain (Online Resource 1).

### Structure of the firefly stemmata

**Fig. 2:**
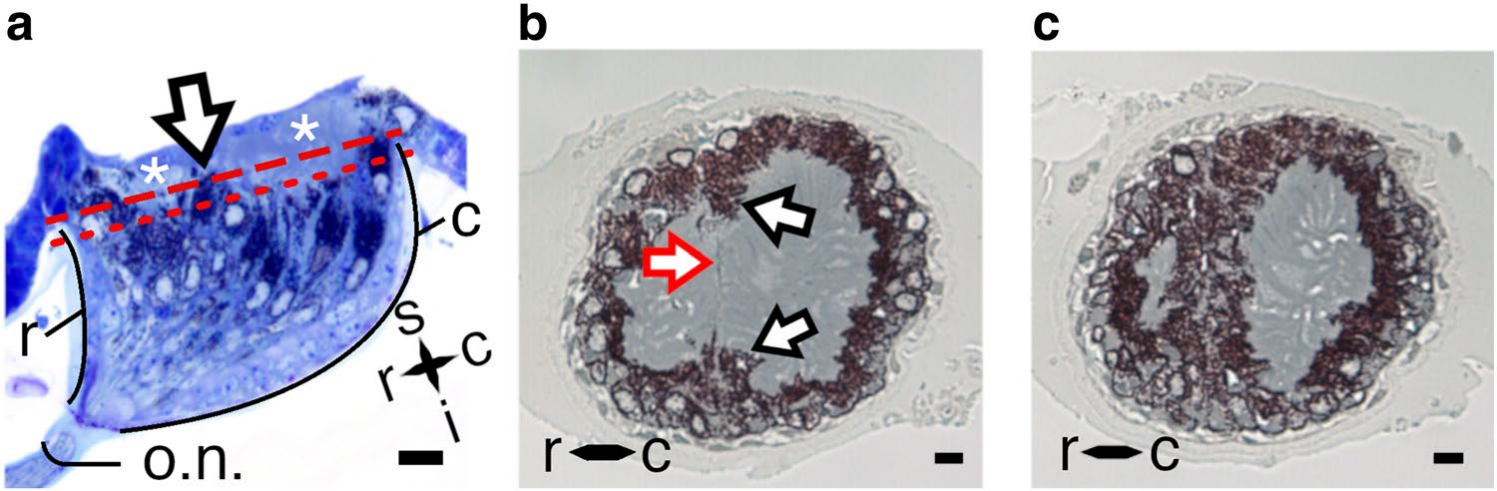
Bilobed structure of the firefly stemmata. **a** Longitudinal section (2 µm thick) counterstained with azure II methylene blue. Two lobes which lack pigment, at the superior aspect of the eye are indicated by asterisks. Pigment granules within a septum (arrow) partition each lobe. Red lines indicate the location of the cross-sections displayed in ‘b’ (superior red dashed line) and ‘c’ (inferior red dotted line), respectively. Black curved lines indicate the exterior curved surfaces of the eye, labeled *r* and *c* for anatomical reference. Scale = 17 µm **b, c** 2-µm cross-sections of stemmata stained with ethyl gallate. **b** Interface of the two lobes is indicated by the red outlined arrow. Protruding structures from the surrounding pigment (black outlined arrows) form a septum. Scale = 9 µm. **c** Pigmented septum ~ 10 µm inferior to the location of the cross-section shown in ‘b.’ Scale = 8.5 µm. *s* superior, *i* inferior, *r* rostral, *c* caudal, *o.n*. optic nerve. The lens is not present in these sections

The longitudinal section of the eye contained dense pigmentation (Fig. 2a). Two regions that lacked pigment granules were conspicuous at the superior surface of the stemmata (asterisks, Fig. 2a). We referred to these two regions as *lobes*, which we defined as independent regions within the stemmata that did not contain pigment granules. In all stemmata studied (*n* = 8) the two lobes were asymmetric. The larger lobe was contiguous with the more prominently curved caudal surface of the eye, in the longitudinal plane (black curved line ‘c,’ Fig. 2a). The smaller lobe was bordered by the comparatively less curved rostral surface of the eye (black curved line ‘r,’ Fig. 2a). The rostral and caudal designations used to describe the stemmata anatomy were defined relative to the position of each lobe beneath the lens in the dissected animal (see Online Resource 2). Dense pigment granules surrounded the rostral, caudal and inferior surfaces of each lobe in a hemispherical pattern and a narrow band of pigmentation was observed between each lobe (arrow, Fig. 2a), suggesting that the lobes were separated structures. In cross-section, it was apparent that the lobes were separated by a pigmented septum. The septum fully separated the lobes at their base forming two distinct asymmetric lobes (Fig. 2b, c).

### The optic nerve

**Fig. 3:**
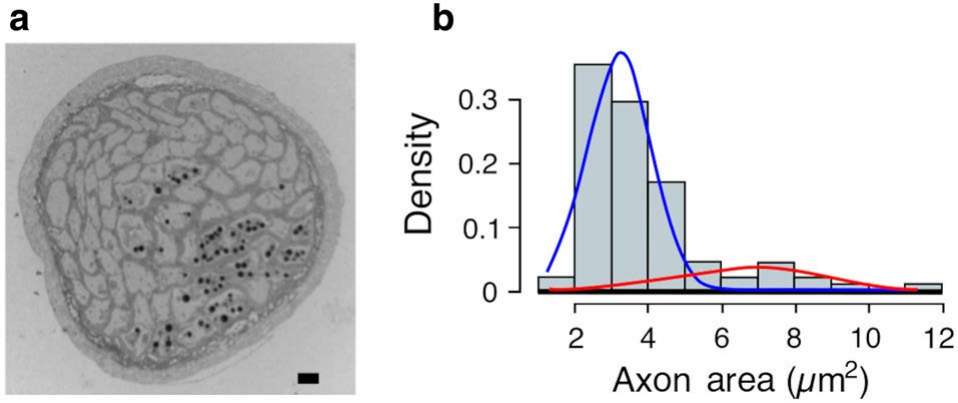
Optic nerve. **a** Cross-section of the optic nerve and it’s axons (*n* = 88). **b** Histogram of the optic nerve areas of all 88 photoreceptor axons measured in ‘a’. This distribution is consistent with there being two size populations (see text for details). The area under each curve (lambda) indicates the mixture densities (blue curve lambda ~ 80%, red curve lambda ~ 20%)

The number of axons within the optic nerve was counted from a cross-section of the optic nerve acquired ~ 5 µm inferior to the base of the stemmata (opposite the lens) where all axons could be traced directly to the eye (Fig. 3). The optic nerve contained 88 axons (± 0.836, *n* = 4) (Fig. 3a). The areas of all 88 axons were measured (histogram, Fig. 3b). The distribution of axonal areas was not consistent with a uniform distribution around a single mean (Anderson–Darling Statistic 4.289, *p* < 0.05). Mclust (a contributed R package for Gaussian decomposition) attributed the distribution into two populations. Using Mixtools the means of these two populations were 3.2 µm^2^ and 6.7 µm^2^, accounting for ~ 80% and ~ 20% of the population, respectively (Fig. 3b).

### Rhabdomere ultrastructure

**Fig. 4:**
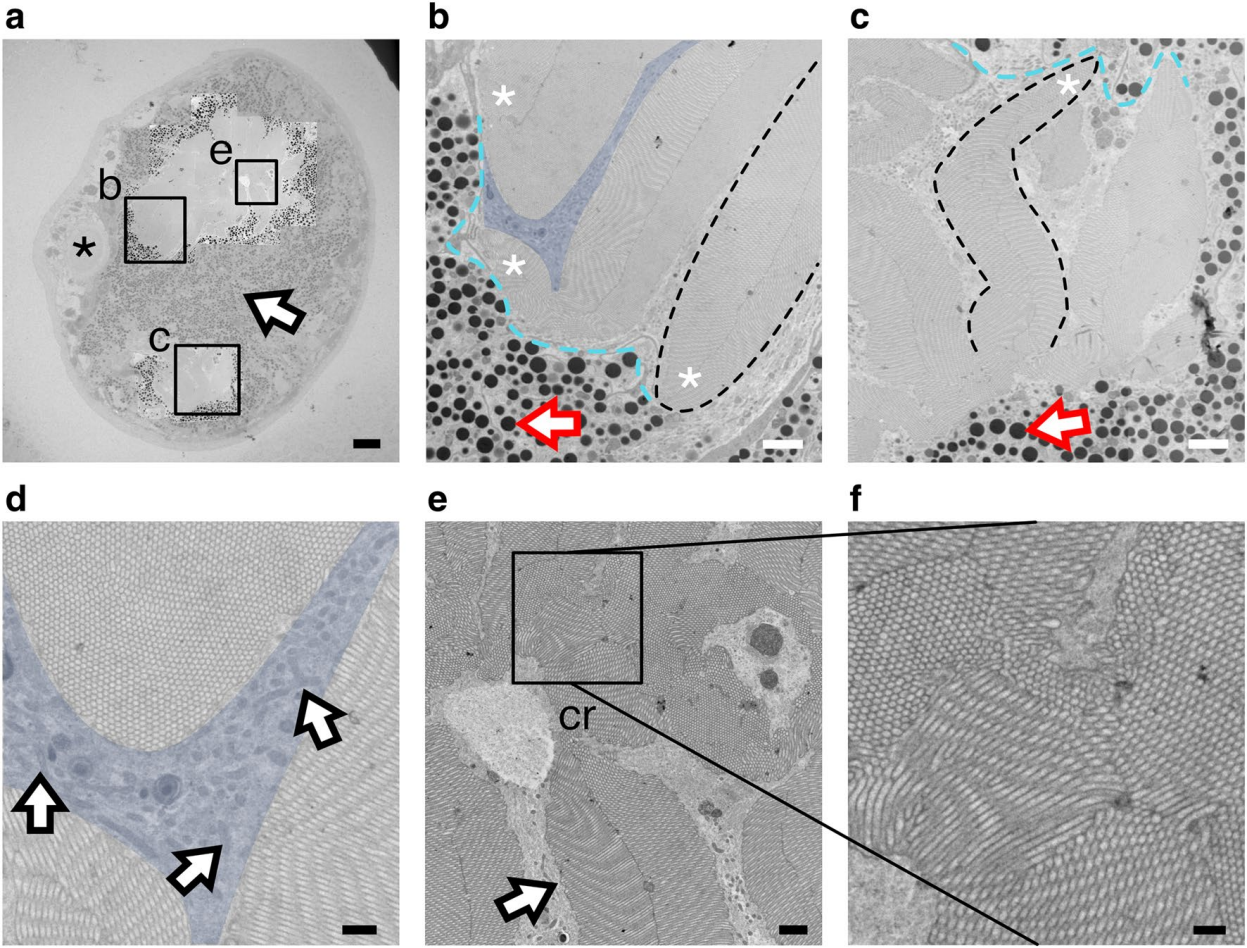
Ultrastructure of the rhabdoms. **a** Cross-sectional overview of a stemmata. Regions of the large and small rhabdoms have been emphasized by reducing the contrast of the background. The septum separating the two rhabdoms is indicated by the arrow. A fragment of the lens is marked by an asterisk. Scale = 10 µm. **b, c** Magnified region of rhabdomeres from the large (**b**) and small (**c**) rhabdom, boxed in ‘a.’ Three rhabdomere groups, with their peripheral regions indicated by the asterisks. A single rhabdomere is outlined with the black dashed line. The inter-rhabdomeric space is indicated by the blue shaded region (b only). Individual pigment granules are marked by arrows. The ‘periphery’ of the rhabdom (i.e., the interface between the apical region of rhabdomeres and the pigment granules) is demarcated by the cyan dashed line. Scale ‘b’ = 2 µm; Scale ‘c’ = 1 µm. **d** Magnified region of inter-rhabdomeric space shaded blue from ‘b.’ Mitochondria indicated by arrows. Scale = 500 nm. **e** Central area of the large rhabdom, region indicated in ‘a.’ A rhabdomere extending into the central region from the periphery (arrows). ‘cr’ = central region. Scale = 1 µm **f** Selection of the central region of large rhabdom magnified from ‘e.’ Scale = 500 nm

Stemmata were cut in cross-section at an orientation that approximated the light microscopy sections featured in Fig. 2b, c. The two regions we referred to as lobes at the light microscope level were identified as two independent rhabdoms, separated by a pigmented septum (arrow, Fig. 4a). Within each rhabdom were multiple finger-like projections of rhabdomeres, wherein microvilli, the sites of insect photoreception, were located. In each cross-section, both rhabdoms contained dense packing of rhabdomeres and were devoid of pigment granules (Fig. 4a; Online Resource 3).

The *periphery* of each rhabdom was defined in cross-section as the interface of rhabdomeres and the surrounding pigment granules (cyan dashed line, Fig. 4b, c). At the periphery, rhabdomeres were sparsely packed. Regions of tissue devoid of rhabdomeres and pigment, but rich in cellular organelles, such as mitochondria, surrounded the rhabdomere tips (arrows, Fig. 4d). We referred to these regions as inter-rhabdomeric space (blue shading, Fig. 4b, d). Moving away from the periphery and towards the center of each rhabdom, the inter-rhabdomeric space became less pronounced; here, the rhabdom was composed of densely packed, interconnected rhabdomeres (Fig. 4e, f).

**Fig. 5:**
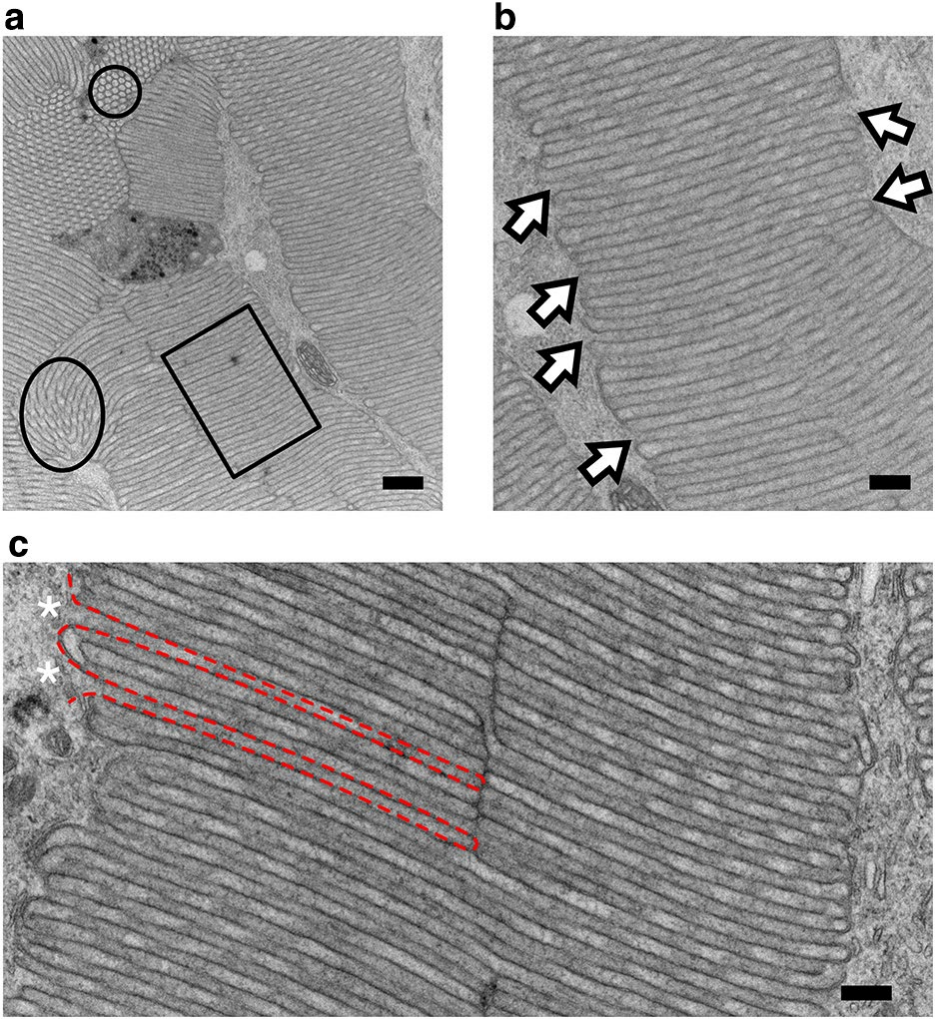
Microvilli. **a** Microvilli orientations. Longitudinal (rectangle), transverse (circle) and intermediate (oval) orientations. Scale = 1 µm. **b** Cross-section through a rhabdomere with microvilli captured in the longitudinal plane. The base of a single microvillus that shares an intracellular compartment with its neighboring inter-rhabdomeric space indicated by arrows. Each single arrow is labeling a single, different microvillus. Scale = 250 nm. **c** Border of individual microvilli. The border surrounding two microvilli is traced by the red dashed line. The areas marked by the asterisks share an intracellular compartment with the adjacent inter-rhabdomeric space and are enclosed by a membrane at the other end. Scale = 250 nm

In all cross-sections containing rhabdomeres, the microvilli, specifically near the center of the rhabdom, exhibited complex multi-directional orientations. An example of this is shown in Fig. 4f. Microvilli were divided into three classes based on their orientation in cross-sections of the stemmata: longitudinal, which produced long slender microvillar tubes (rectangle, Fig. 5a), transverse, indicated by the circular profiles of microvilli (circle, Fig. 5a) and intermediate orientations (between longitudinal and transverse) that resulted from microvilli passing through the plane of section at different angles or through the bending of microvillus folds (oval, Fig. 5a). Microvilli coplaner with the plane of section were repeatedly observed with intracellular domains contiguous with neighboring inter-rhabdomeric space (arrows indicated the shared intracellular compartment at the base of an individual microvillus Fig. 5b). Microvilli captured in the longitudinal orientation were enclosed by a membrane around all surfaces except at the interface between the base of an individual microvillus and the inter-rhabdomeric space (base indicated by asterisk, borders indicated by red dashed outline, Fig. 5c).

**Fig. 6:**
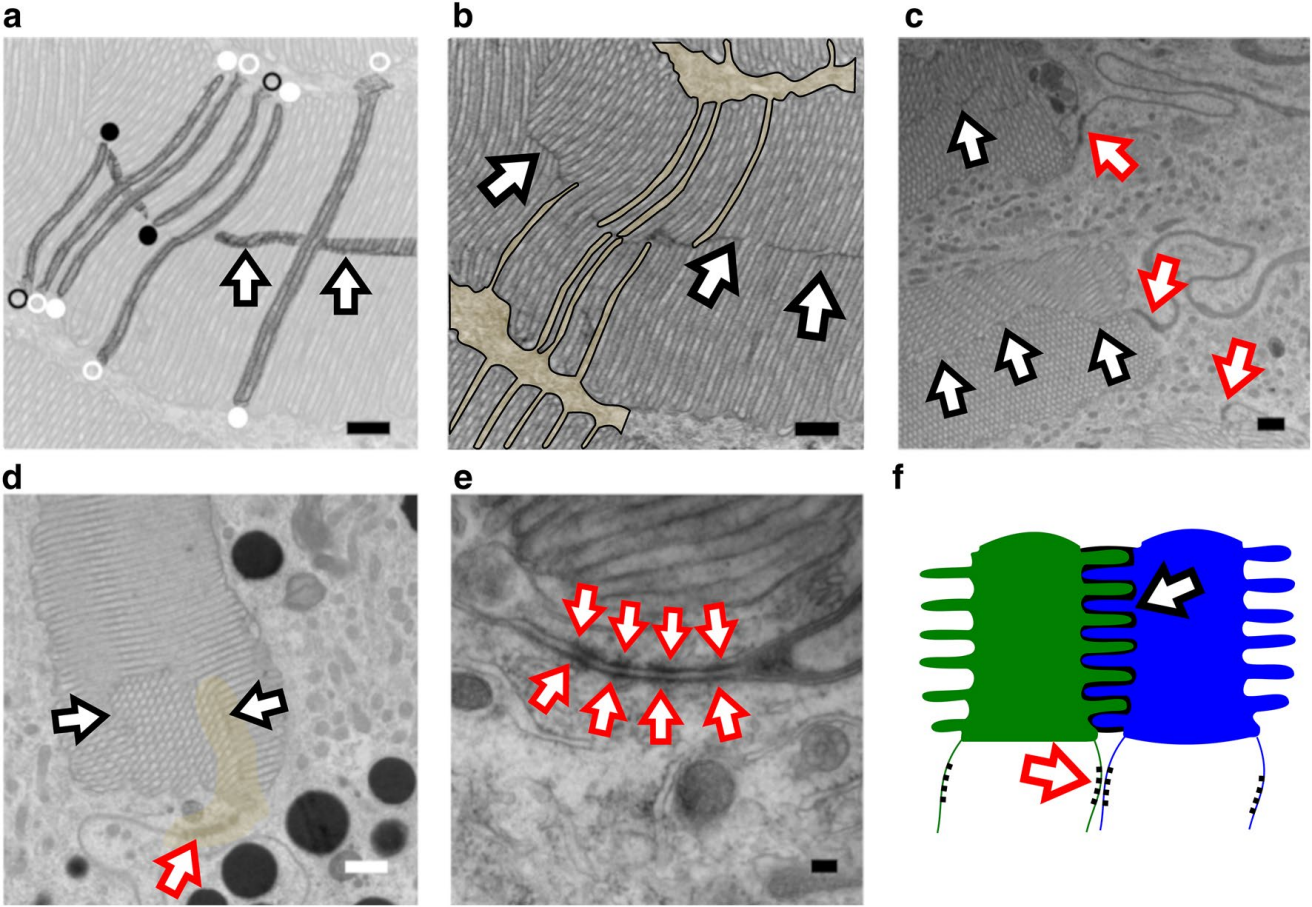
Ultrastructure of the photoreceptor cell body and its rhabdomeres. **a** Some microvilli appeared to extend the width of the rhabdomere, indicated by white circles. Other microvilli within the rhabdomere appeared shorter, (black circles). The end of the microvillus that shares an intracellular compartment with its adjacent inter-rhabdomeric space is marked by unfilled circles (regardless of color). The ‘enclosed’ end of microvilli is indicated by filled circles (regardless of color). An electron-dense, bisecting structure that sporadically appears within a single cross-section is indicated by arrows. Contrast of the image is reduced with the exception of the labelled microvilli. Scale = 200 nm. **b** A single inter-rhabdomeric space shares an intracellular compartment with adjacent microvilli. Same image pictured in ‘a,’ with un-modified contrast. Select inter-rhabdomeric space is surrounded by the black outline and filled with tan shading. Electron-dense septal structure is indicated by arrows. Scale = 200 nm. **c** Electron-dense, septal structure within rhabdomeres, indicated by black outlined arrows. Scale = 500 nm. **d** Membrane structure extending off the exterior portion of the peripheral region of a rhabdomere. Membrane structure interior and exterior to the rhabdomere periphery is indicated with tan shading. Black outlined arrows indicate septal structure within the rhabdomere. Scale = 500 nm. **e** Adherens junctions. 4 pairs of adherens junctions indicated by the red outlined arrows. Scale = 100 nm. Red outlined arrows pictured in ‘c, d’ point to adherens junctions at lower magnification. **f** Schematic of two photoreceptor cells (green and blue). A stylized, single rhabdomere pair with interdigitating microvilli separated by a thin electron-dense membrane (black shading). Black outlined arrow indicates a single microvillus sharing intracellular space with the blue cell. Adherens junctions are indicated by the red outlined arrow

An additional finding was that microvilli were unique to only one inter-rhabdomeric space (Fig. 6). In some instances, microvilli extended the width of the rhabdomeres until the individual microvillus was enclosed by a surrounding membrane (filled white circles, Fig. 6a). In other cases, microvilli did not extend the full width of the rhabdomeres (filled black circles, Fig. 6a). When microvilli did not extend the full width of the rhabdomeres, a thin electron-dense structure, presumably a membrane, was visible (arrows, Fig. 6a, b). This may reflect an inherent limitation in the ultrathin EM sections. Microvilli passing through the plane of section at an intermediate orientation will appear to terminate in spite of the fact they may extend the full length of the rhabdomere. Among those microvilli that extended the full length, in no case did we find a microvillus whose intracellular compartment was shared between multiple inter-rhabdomeric cells (Fig. 6a, b). In contrast, we found many instances of individual inter-rhabdomeric cells contiguous with the intracellular compartments of adjacent microvilli in different directions (black outline, Fig. 6b). We suggest that this is consistent with a model of interdigitating microvilli from neighboring inter-rhabdomeric cells (Fig. 6f).

Multiple pairs of adherens junctions couple the membranes of adjacent photoreceptors (red outlined arrows, Fig. 6c–e). These junctions were apparently independent of microvillar orientation (Fig. 6c, d). These data suggested that rhabdomeres extending from the periphery towards the center of the rhabdom were organized as pairs of neighboring photoreceptors whose microvilli were interdigitated as depicted schematically in Fig. 6f.

### Rhabdom organization

**Fig. 7:**
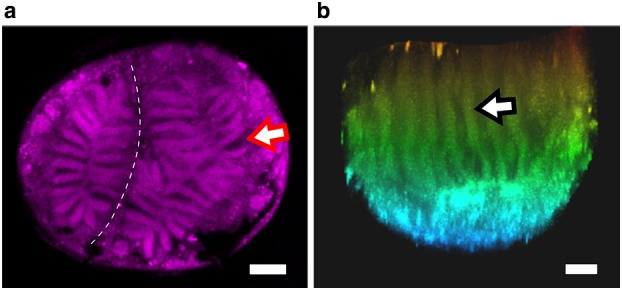
Autofluorescent confocal microscopy of rhabdomeres. **a** Radial organization of large and small rhabdoms in an optical cross-section through the stemmata. The pigmented septal region that separates the two rhabdoms is indicated by the white dashed line. A single example of the inter-rhabdomeric space, characterized by lack of fluorescence, is indicated by the arrow. Scale = 25 µm. **b** Vertical, columnar, organization of rhabdomeres extending the depth of the small rhabdom. A single rhabdomere column is indicated by the arrow. The image is depth coded to accentuate the vertical orientation of rhabdomeres. Orange = superior aspect of the eye, nearest the lens, blue = inferior aspect of the eye nearest the optic nerve. Scale = 25 µm

Confocal microscopy was used to put the emerging structure of the eye, i.e., the size, shape and location of the rhabdomeres, into a three-dimensional context. Rhabdomeres were autofluorescent. Using an excitation wavelength of 561 nm, strong autofluorescence was viewed with an acceptance range of 590–630 nm (Fig. 7). Radial rhabdomere pairs from the periphery extended inward and converged on a common central region (Fig. 7a). Rhabdomeres, specifically at the periphery of the rhabdoms, were flanked by non-fluorescent regions whose location and organization were consistent with the photoreceptor cell bodies previously identified in Figs. 4, 5 and 6 (arrow, Fig. 7a). Rhabdomeres projected the depth of each rhabdom in a columnar fashion (Fig. 7b). In the longitudinal plane of the stemmata, columns were organized vertically (arrow, Fig. 7b).

### Photoreceptor structure

**Fig. 8:**
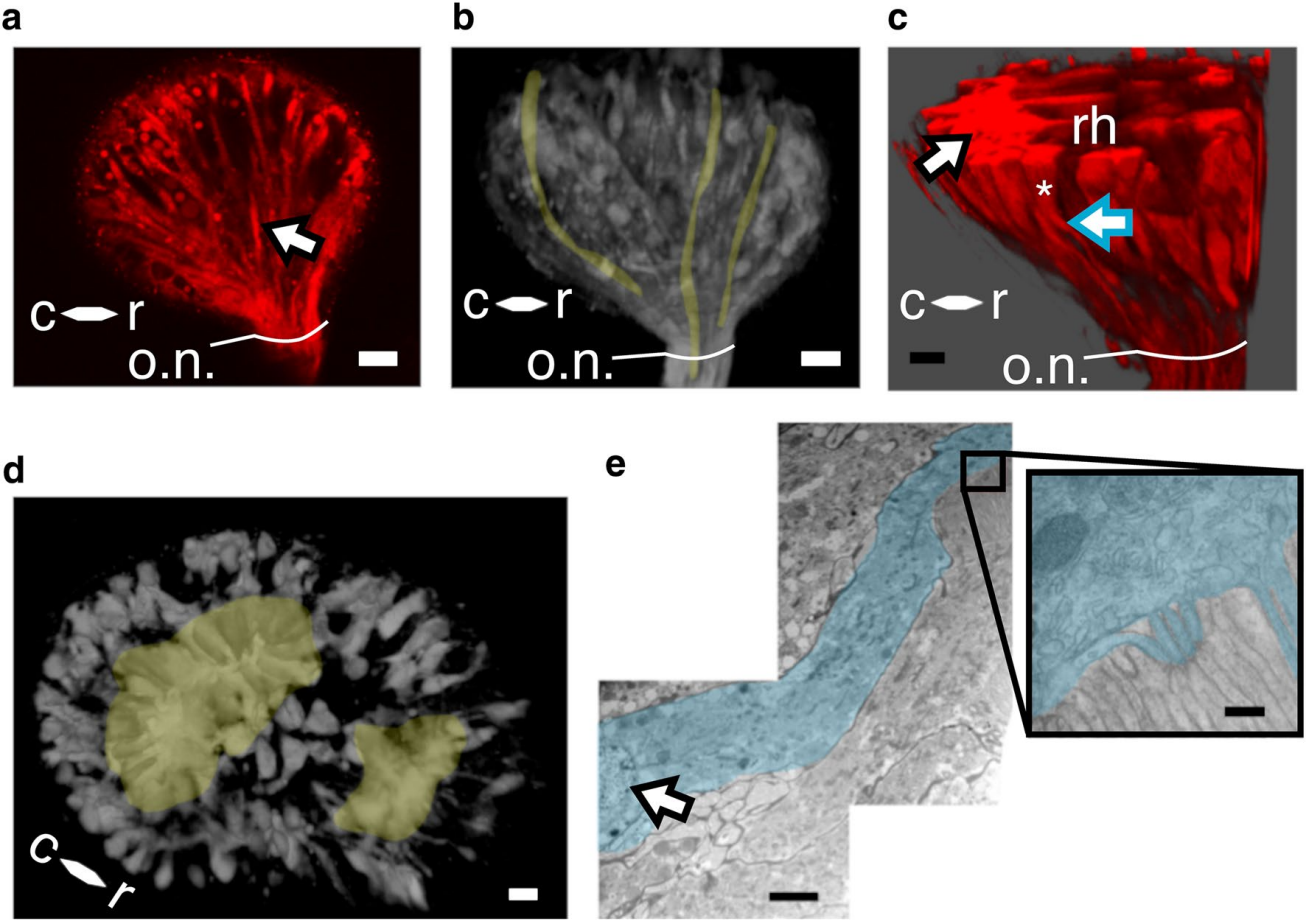
Photoreceptors. **a** Texas-Red filled axons. Scale = 15 µm. **b** Three axons are highlighted in tan to facilitate visualization of the optic nerve along the exterior curvature of the eye. Scale = 15 µm. **c** Three-dimensional projection of photoreceptors lining the exterior of the eye. A single axon emerging from the base of a photoreceptor cell body is marked by the blue outlined arrow. A single photoreceptor cell body is indicated by an asterisk. A collection of photoreceptors surrounding the large rhabdom is indicated by the black outlined arrow. A rhabdomere pair extending from photoreceptors, composing the large rhabdom, is labeled ‘rh.’ Scale = 10 µm. **d** Bilobed structure. Large and small rhabdoms highlighted in tan surrounded by photoreceptor cells. Perspective is looking into the eye through the lens. Scale = 10 µm. **e** Ultrastructure of a single photoreceptor. A single photoreceptor is shaded in blue with the nucleus identified by the black outlined arrow. Scale = 2 µm. The photoreceptor cell body merging into the microvilli is shaded in blue and shown in the boxed region. Unshaded region = microvilli of the adjacent, interdigitating rhabdomere pair. Scale = 100 nm. *o.n*. optic nerve, *c* caudal, *r* rostral

The autofluorescence was sufficient to visualize the organization of the rhabdomeres within each rhabdom, but did not elucidate the morphology of the photoreceptor cell bodies or their connection to the optic nerves. Cell bodies were visualized with Texas Red backfills of optic nerve axons (black outlined arrow, Fig. 8a, tan shading, Fig. 8b). Photoreceptors were conical in shape and distinguishable as individual cells arranged in vertical columns along the exterior surfaces of the eye surrounding the rhabdoms (asterisk, Fig. 8c). Surrounding the large rhabdom, the photoreceptors were tightly packed (black outlined arrow Fig. 8c, tan shading Fig. 8d) which produced a singular dense fluorescent profile where we were unable to tease apart individual cells. Rhabdomeres extended from the dense region of photoreceptors contributing the large rhabdom (‘rh’, Fig. 8c). Axons projected from the base of the photoreceptor cell bodies to the optic nerve (tan shading, Fig. 8b, blue outlined arrow Fig. 8c). Consistent with the TEM and the autofluorescence images of the rhabdoms, the rhabdomeres projected towards the center region of the rhabdom and appeared to extend the depth of the eye, in the direction of the optic nerve, as a solid ‘sheet’ (Fig. 8c ‘rh’). A single photoreceptor, identified by its nucleus and conical shape, gave rise to microvilli extending from a single portion of the cell body, proximal to the lens (Fig. 8e). Here, the microvilli were shown in interlocking fashion with microvilli from its neighboring photoreceptor (boxed region, Fig. 8e). The three-dimensional structure of the entire stemmata is included as a rotating image in the online resources (Online Resource 4).

## Discussion

Fireflies are holometabolous, polyphagous beetles. *Photuris* adults possess large (1 mm) compound eyes on the ventral surface of the head (Horridge 1969), with multiple optic ganglia (Strausfeld and Blest 1970). These neural superposition eyes and underlying visual centers have been shown to be critical for the acquisition and processing of conspecific bioluminescent flash patterns during courtship and mating behavior (Carlson and Copeland 1985). Firefly larvae do possess a visual system, and are bioluminescent, but do not manifest the same visually mediated behaviors as adults. We found that, consistent with other polyphagous beetles, firefly larvae had single-lensed stemmata positioned on the lateral surfaces of the head (Fig. 1b). Each stemma produced an optic nerve which projects directly to the protocerebrum, indicating an absence of any optic ganglia external to the brain (Online Resource 1). This indicates a direct pathway for visual information between the eye and the brain. As a first order to understanding the putative ecological utility and/or physiological function of the larval visual system, we examined the gross microscopic structure of the stemmata and found dual rhabdoms beneath the singular lens. Ultrastructural analysis of each lobe reveals that rhabdomeres extended from the periphery of each rhabdom into a shared central region, indicating a fused net-like rhabdom (Snyder et al. 1973) (Fig. 7).

### Dual-rhabdom, fusion stemmata

Within and across the four major orders of holometabola, zero to seven stemmata are found in bilateral organization on the larval head, where the precise number of stemmata is contingent upon its phylogenetic classification (Gilbert 1994). Ancestrally, beetles (Coleoptera) have six stemmata on each side of their head (Paulus 1986). Yet, not all beetles have six eyes. This is attributed to independent evolutionary processes (loss and/or reduction of stemmata). Despite heterogeneity in number, stemmata have evolved from ommatidia, the individual units that compose the compound eye (Paulus 1986; Friedrich et al. 2011; Buschbeck 2014). Thus, many stemmata bear cellular resemblance to adult ommatidia. Firefly stemmata did not adhere to the canonical ommatidial architecture; neither in terms of organization or photoreceptor number (Figs. 3, 4, 5). Instead, the stemmata had two, differently sized rhabdoms (Figs. 2, 7; Online Resource 3) that were anatomically separate. The rhabdomeres within each rhabdom were ‘wide’ and organized in pairs at the periphery (Fig. 5), comprising the majority of the cross-sectional surface in the sections displayed (Figs. 2b, c; 7a, b). By contrast, in ommatidia, the rhabdoms are narrow and long, not wide and stout (Buschbeck 2014). Furthermore, while the rhabdoms had a general column-like organization (Fig. 7), the rhabdomeres and the microvilli they contained did not conform to a uniform directionality (multi-plane curvature of microvilli, Figs. 4c, e, f and 5a). Furthermore, *Photuris* firefly larvae have more photoreceptors, 88 (Fig. 3a), than the ancestral ommatidial body plan for beetles would suggest, 48 (6 stemmata and 8 photoreceptors per ommatidia). Taken together, this structure is consistent with fusion stemmata (Paulus 1986, 2000; Gilbert 1994), where two or more units of ommatidia fuse together beneath a single lens.

The fused organization of the firefly stemmata resembled the asymmetrically sized fusion stemmata of *Tribolium castaneum*. In *T. castaneum* larvae, another polyphagous beetle, the stemmata have two clusters of retinula cells that form dorsal and ventral lobes. The asymmetry in the size of these clusters is attributed to the number of photoreceptors, where the larger cluster contains more retinula cells (Liu and Friedrich 2004). It is unknown how the firefly stemmata are formed.

### Axon populations in the optic nerve

The optic nerve contained axons of multiple sizes (Fig. 3a) divided into two discrete populations based on axon size (Fig. 3b). Given that there are two distinct regions of the eye, i.e., two rhabdoms, we hypothesize that the two size populations innervate the two lobes, respectively. This may suggest that populations of retinula cells have different properties specialized for different function (e.g., different thresholds for detecting light or different rhodopsin populations). Determining the underpinnings of the anatomical circuit of first-order visual neurons and probing for the putative functional capacity of each lobe will provide insight into the visual landscape of a firefly larva.

### Ecological implication

Wide, net-like rhabdomere pairs arranged in vertical columns with a shared central region indicate that each rhabdom has a ‘fused’ organization. This is consistent with rhabdoms that are optimized for maximal photon capture (Snyder et al. 1973). It is unlikely the eye is designed for polarized light reception given the non-uniform direction of microvilli throughout the eye (Labhart and Meyer 1999). These data suggest firefly larval eyes may be optimized for vision in nocturnal environments. It is known that firefly larvae are active at night (McLean et al. 1972). Nocturnal activity of firefly larvae such as bioluminescent glowing and locomotory behavior has been observed in the animal’s natural ecosystem. To our knowledge, firefly larvae have not been observed in the field during natural daylight. As a corollary, the bioluminescence produced by fireflies and other beetle larvae are strongly linked to a defense mechanism (Sivinski 1981; Underwood et al. 1997). Considering that bioluminescence in larvae is an aposematic display, we hypothesize that glowing would be most effective in dim lit environments where contrast of the bioluminescence would facilitate other individuals seeing the bioluminescent glow. These field correlations in tandem with our structural observations of rhabdomere configurations in the eye necessitate the exploration of stemmata function in nocturnal conditions.

Structure and function of single-lensed stemmata systems, specifically in elaterids, is not well known. Despite many polyphagous beetles displaying a single-lens larval visual system, convergent evolution and independent adaptations governed by environmental pressures have undoubtedly produced unique optical strategies throughout holometabolous larvae. Fireflies, known for their bioluminescence and adult visual behaviors, spend the majority of their lives in larval form with a stemmata visual system. In a broader perspective, we believe fireflies can be used as a viable model to study how sensory systems are tuned for ecological functions.

## Electronic supplementary material

Below is the link to the electronic supplementary material.

Supplementary material 1. **Fig. S1**. Gross anatomy of firefly larval central nervous system. Stemmata (black outlined arrows) and their optic nerves (red outlined arrow) project caudally into a nerve bundle containing two additional nerve fibers. The merged nerve bundle indicated by black ovals projects directly into the protocerebrum (blue outlined arrow). *a.n.f*. additional nerve fibers, *r* rostral, *c* caudal. Scale = 250 µm (PDF 921 KB)

Supplementary material 2. **Fig. S2**. In situ orientation of the rhabdoms of stemmata. Looking down on the dorsal surface, the cuticle is removed revealing a single stemmata. The large rhabdom (black outlined arrow) is positioned caudally to the small rhabdom (red outlined arrow). The mouth parts (*mp*) indicate the rostral position of the larval head. *c* caudal, *r* rostral, *m* medial, *l* lateral. Scale = 100 µm (PDF 1503 KB)

Supplementary material 3. **Fig. S3**. Large and small rhabdom. **a**, **b** Micrograph montage of the large and small rhabdom pictured in Fig. 4. Scale = 2 µm (PDF 1681 KB)

Supplementary material 4. **Fig. S4**. Video of a single three-dimensional reconstructed stemmata. The video begins with the exterior surface of the eye. Axons and cell bodies are visible. The stemmata rotates in the vertical plane, exposing the surface of the eye as viewed through the lens (here the large and small lobes are visible, use Fig. 8d for reference). The horizontal field of view is ~ 180 µm in length (MOV 39171 KB)

## References

[CR1] Benaglia T, Chauveau D, Hunter DR, Young DS (2009). mixtools: an R package for analyzing finite mixture models. J Stat Softw.

[CR2] Briscoe AD, Chittka L (2001). The evolution of color vision in insects. Annu Rev Entomol.

[CR3] Buschbeck EK (2014). Escaping compound eye ancestry: the evolution of single-chamber eyes in holometabolous larvae. J Exp Biol.

[CR4] Buschbeck EK, Sbita SJ, Morgan RC (2007). Scanning behavior by larvae of the predacious diving beetle, *Thermonectus marmoratus* (Coleoptera: Dytiscidae) enlarges visual field prior to prey capture. J Comp Physiol A.

[CR5] Carlson AD (1968). Effect of adrenergic drugs on the lantern of the larval *Photuris* firefly. J Exp Biol.

[CR6] Carlson AD, Copeland J (1985). Flash communication in fireflies. Q Rev Biol.

[CR7] Farnier K, Dyer AG, Taylor GS, Peters RA, Steinbauer MJ (2015). Visual acuity trade-offs and microhabitat-driven adaptation of searching behaviour in psyllids (Hemiptera: Psylloidea: Aphalaridae). J Exp Biol.

[CR8] Fraley C, Raftery AE (2007). Model-based methods of classification: using the mclust software in chemometrics. J Stat Softw.

[CR9] Friedrich M, Wood EJ, Wu M (2011). Developmental evolution of the insect retina: insights from standardized numbering of homologous photoreceptors. J Exp Zoolog B Mol Dev Evol.

[CR10] Gilbert C (1994). Form and function of stemmata in larvae of holometabolous insects. Annu Rev Entomol.

[CR11] Harzsch S, Müller CH, Wolf H (2005). From variable to constant cell numbers: cellular characteristics of the arthropod nervous system argue against a sister-group relationship of Chelicerata and “Myriapoda” but favour the Mandibulata concept. Dev Genes Evol.

[CR12] Horridge GA (1969) The eye of the firefly *Photuris*. Proc R Soc Lond B Biol Sci 445–463

[CR13] Katsov AY, Clandinin TR (2008). Motion processing streams in drosophila are behaviorally specialized. Neuron.

[CR14] Kristensen NP (1999). Phylogeny of endopterygote insects, the most successful lineage of living organisms. Eur J Entomol.

[CR15] Labhart T, Meyer EP (1999). Detectors for polarized skylight in insects: a survey of ommatidial specializations in the dorsal rim area of the compound eye. Microsc Res Tech.

[CR16] Land MF (1997). Visual acuity in insects. Annu Rev Entomol.

[CR17] Leise EM, Mulloney B (1986). The osmium-ethyl gallate procedure is superior to silver impregnations for mapping neuronal pathways. Brain Res.

[CR18] Liu Z, Friedrich M (2004). The *Tribolium* homologue of glass and the evolution of insect larval eyes. Dev Biol.

[CR19] Mandapaka K, Morgan RC, Buschbeck EK (2006). Twenty-eight retinas but only twelve eyes: an anatomical analysis of the larval visual system of the diving beetle *Thermonectus marmoratus* (Coleoptera: Dytiscidae). J Comp Neurol.

[CR20] McLean M, Buck J, Hanson FE (1972) Culture and larval behavior of photurid fireflies. Am Midl Nat 133–145

[CR21] Melzer RR, Paulus HF, Kristensen NP (1994). The larval eye of nannochoristid scorpionflies (Insecta, Mecoptera). Acta Zool.

[CR22] Melzer RR, Diersch R, Nicastro D, Smola U (1997). Compound eye evolution: highly conserved retinula and cone cell patterns indicate a common origin of the insect and crustacean ommatidium. Naturwissenschaften.

[CR23] Nilsson DE (1989). Vision optics and evolution. Bioscience.

[CR24] Nilsson DE, Kelber A (2007). A functional analysis of compound eye evolution. Arthropod Struct Dev.

[CR25] Paulus HF, Gepp J, Aspock H, Holzel H (1986). Comparative morphology of the larval eyes of Neuropteroidea. Recent research in neuropterology.

[CR26] Paulus HF (2000). Phylogeny of the Myriapoda–Crustacea–Insecta: a new attempt using photoreceptor structure. J Zool Syst Evol Res.

[CR27] Sato T (1968). A modified method for lead staining of thin sections. J Electron Microsc (Tokyo).

[CR28] Schindelin J, Arganda-Carreras I, Frise E, Kaynig V, Longair M, Pietzsch T, Preibisch S, Rueden C, Saalfeld S, Schmid B, Tinevez JY, White DJ, Hartenstein V, Eliceiri K, Tomancak P, Cardona A (2012). Fiji: an open-source platform for biological-image analysis. Nat Methods.

[CR29] Sivinski J (1981) The nature and possible functions of luminescence in Coleoptera larvae. Coleopt Bull 167–179

[CR30] Snyder AW, Menzel R, Laughlin SB (1973). Structure and function of the fused rhabdom. J Comp Physiol.

[CR31] Somanathan H, Borges RM, Warrant EJ, Kelber A (2008). Visual ecology of Indian carpenter bees I: light intensities and flight activity. J Comp Physiol A.

[CR32] Strausfeld NJ, Blest AD (1970). The optic lobes of Lepidoptera. Phil Trans R Soc Lond B.

[CR33] Toh Y, Mizutani A (1994). Structure of the visual system of the larva of the tiger beetle (*Cicindela chinensis*). Cell Tissue Res.

[CR34] Underwood TJ, Tallamy DW, Pesek JD (1997). Bioluminescence in firefly larvae: a test of the aposematic display hypothesis (Coleoptera: Lampyridae). J Insect Behav.

[CR35] Zhang SQ, Che LH, Li Y, Liang D, Pang H, Slipinski A, Zhang P (2018). Evolutionary history of Coleoptera revealed by extensive sampling of genes and species. Nat Commun.

