# Supplementary material for: Anatomy of the stemmata in the Photuris firefly larva
Source: J Comp Physiol A Neuroethol Sens Neural Behav Physiol. 2019 Jan 16;205(1):151–61. doi: 10.1007/s00359-018-01312-2 (PMC6394516; doi:10.1007/s00359-018-01312-2)
Supplement: Supplementary file 1 — Supplementary material 1. Fig. S1. Gross anatomy of firefly larval central nervous system. Stemmata (black outlined arrows) and their optic nerves (red outlined arrow) project caudally into a nerve bundle containing two additional nerve fibers. The merged nerve bundle indicated by black ovals projects directly into the protocerebrum (blue outlined arrow). a.n.f. additional nerve fibers, r rostral, c caudal. Scale = 250 µm (PDF 921 KB) [file 359_2018_1312_MOESM1_ESM.pdf]

Article Title: Anatomy of the Stemmata in the *Photuris* Firefly Larva.

Journal Name: Journal of Comparative Physiology A

Author Names: Fred Murphy, Andrew Moiseff

Affiliation: Department of Physiology and Neurobiology, University of Connecticut

Corresponding Author: Fred Murphy, fred.murphy@uconn.edu

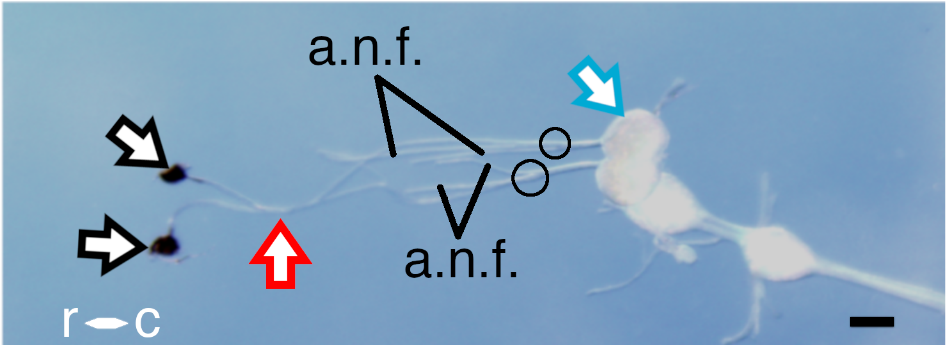
a.n.f.
a.n.f.
r c
