# Supplementary material for: Anatomy of the stemmata in the Photuris firefly larva
Source: J Comp Physiol A Neuroethol Sens Neural Behav Physiol. 2019 Jan 16;205(1):151–61. doi: 10.1007/s00359-018-01312-2 (PMC6394516; doi:10.1007/s00359-018-01312-2)
Supplement: Supplementary file 2 — Supplementary material 2. Fig. S2. In situ orientation of the rhabdoms of stemmata. Looking down on the dorsal surface, the cuticle is removed revealing a single stemmata. The large rhabdom (black outlined arrow) is positioned caudally to the small rhabdom (red outlined arrow). The mouth parts (mp) indicate the rostral position of the larval head. c caudal, r rostral, m medial, l lateral. Scale = 100 µm (PDF 1503 KB) [file 359_2018_1312_MOESM2_ESM.pdf]

Article Title: Anatomy of the Stemmata in the *Photuris* Firefly Larva.

Journal Name: Journal of Comparative Physiology A

Author Names: Fred Murphy, Andrew Moiseff

Affiliation: Department of Physiology and Neurobiology, University of Connecticut

Corresponding Author: Fred Murphy, fred.murphy@uconn.edu

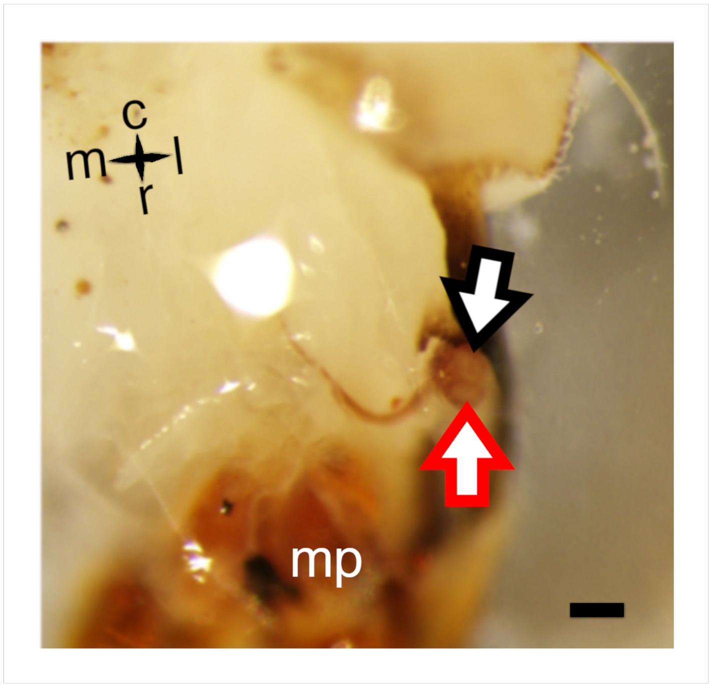
c
m
l
r
mp
