# Supplementary material for: Anatomy of the stemmata in the Photuris firefly larva
Source: J Comp Physiol A Neuroethol Sens Neural Behav Physiol. 2019 Jan 16;205(1):151–61. doi: 10.1007/s00359-018-01312-2 (PMC6394516; doi:10.1007/s00359-018-01312-2)
Supplement: Supplementary file 3 — Supplementary material 3. Fig. S3. Large and small rhabdom. a, b Micrograph montage of the large and small rhabdom pictured in Fig. 4. Scale = 2 µm (PDF 1681 KB) [file 359_2018_1312_MOESM3_ESM.pdf]

Article Title: Anatomy of the Stemmata in the *Photuris* Firefly Larva.

Journal Name: Journal of Comparative Physiology A

Author Names: Fred Murphy, Andrew Moiseff

Affiliation: Department of Physiology and Neurobiology, University of Connecticut

Corresponding Author: Fred Murphy, fred.murphy@uconn.edu

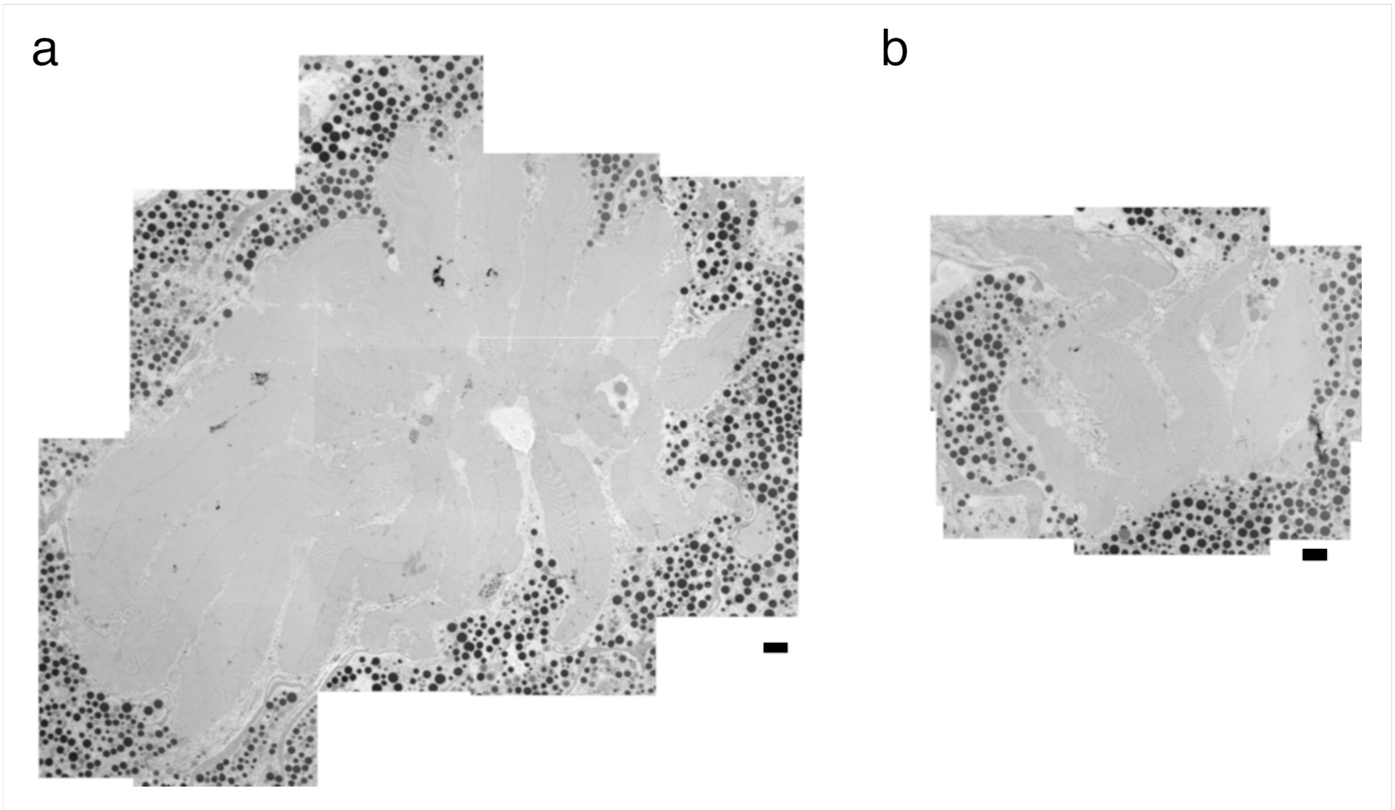
a
b
